# Supplementary material for: Toxoplasma gondii GRA9 Regulates the Activation of NLRP3 Inflammasome to Exert Anti-Septic Effects in Mice
Source: Int J Mol Sci. 2020 Nov 10;21(22):8437. doi: 10.3390/ijms21228437 (PMC7696177; doi:10.3390/ijms21228437)
Supplement: Supplementary file 1 [file ijms-21-08437-s001.pdf]

SUPPLEMENTARY FIGURE and FIGURE LEGENDS

Fig 1A

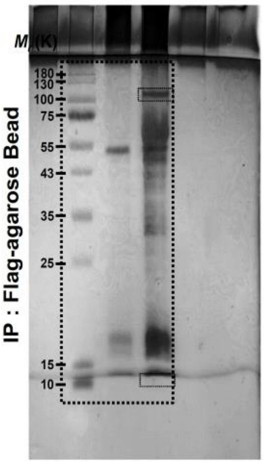

Fig 1B

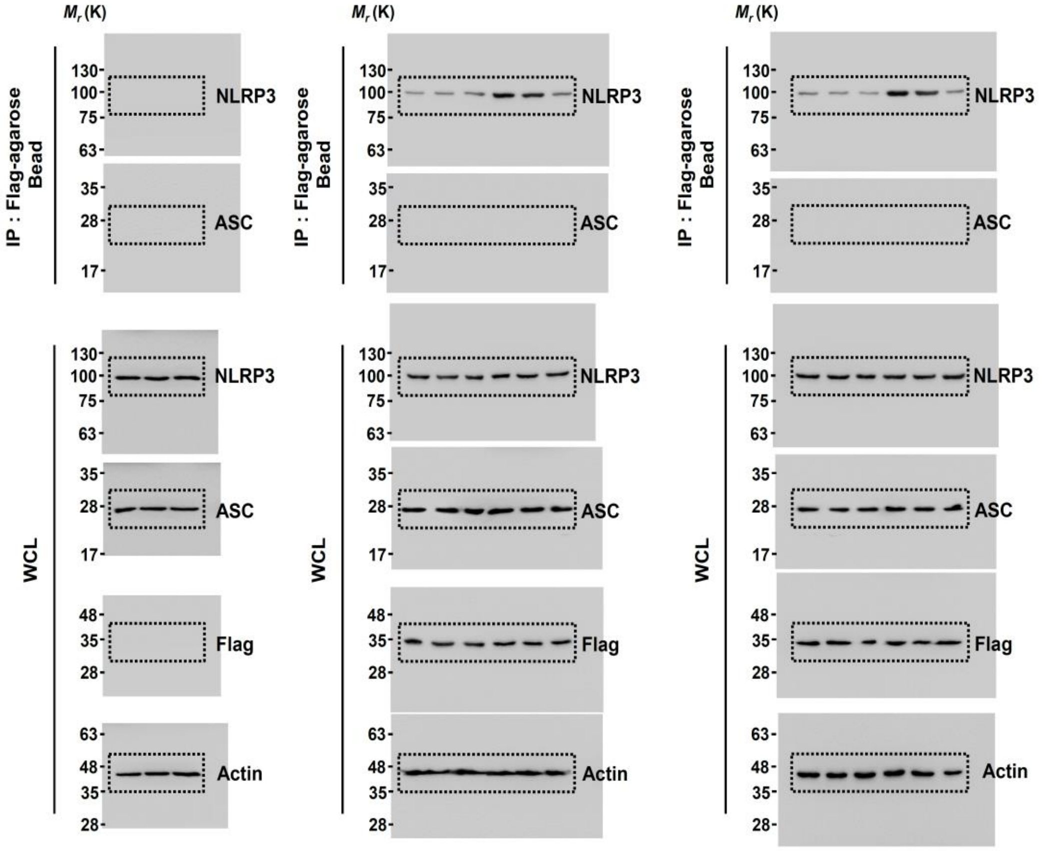

Figure S1. Full-length western blots.

Full-length images of the blots presented in the Figure 1A and 1B.

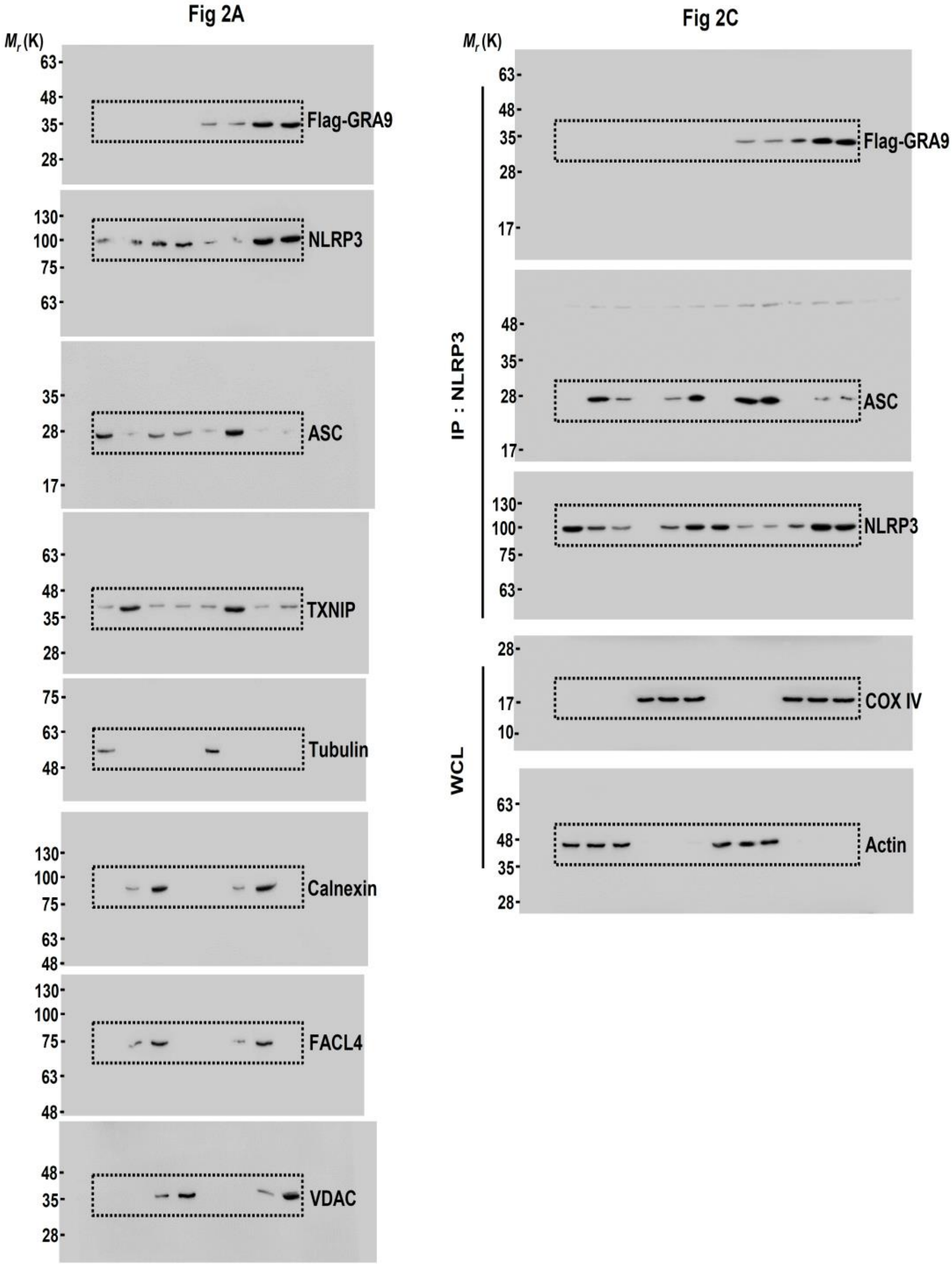

Figure S1. Full-length western blots.

Full-length images of the blots presented in the Figure 2A and 2C.

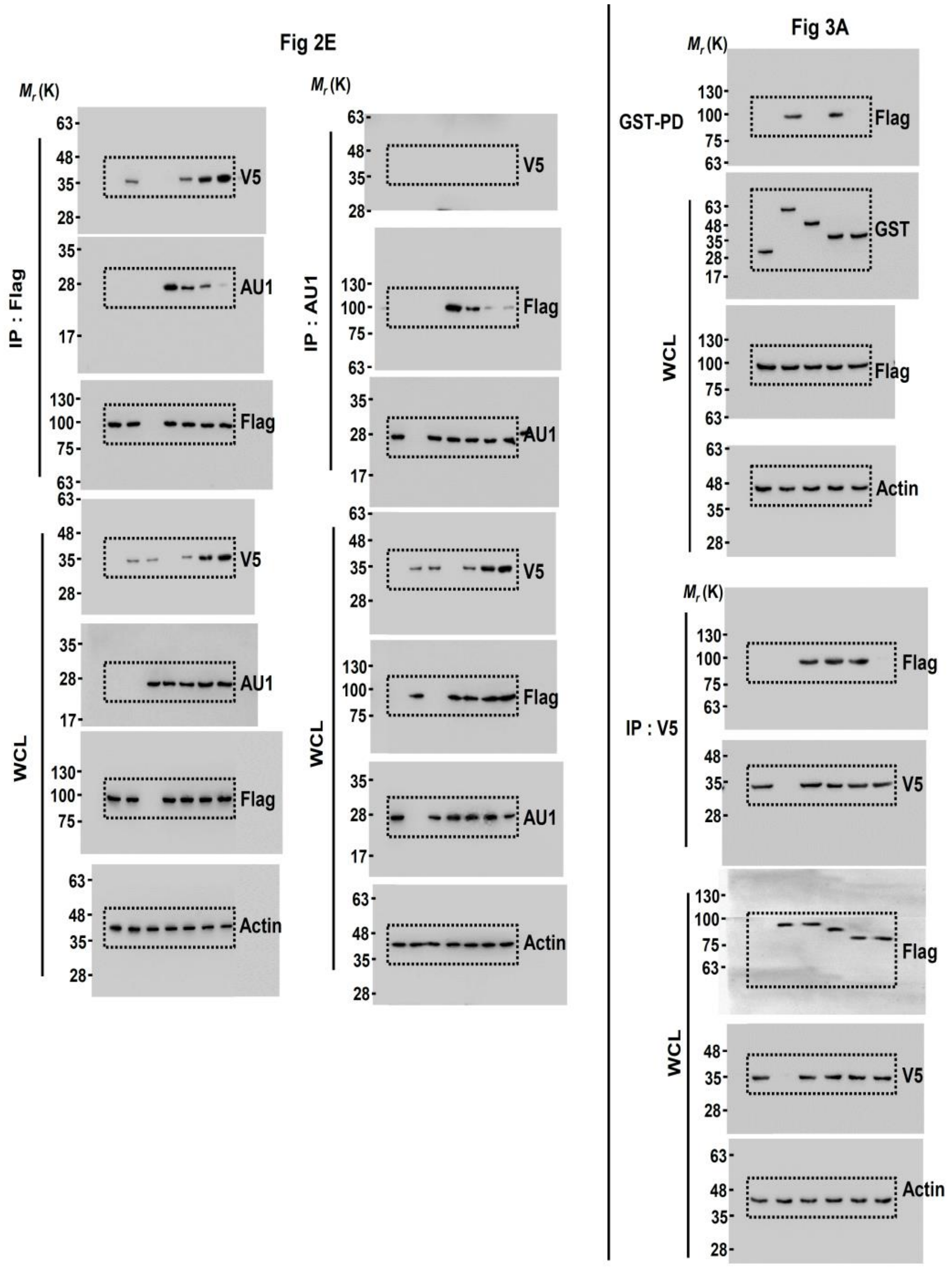

Figure S1. Full-length western blots.

Full-length images of the blots presented in the Figure 2E and 3A.

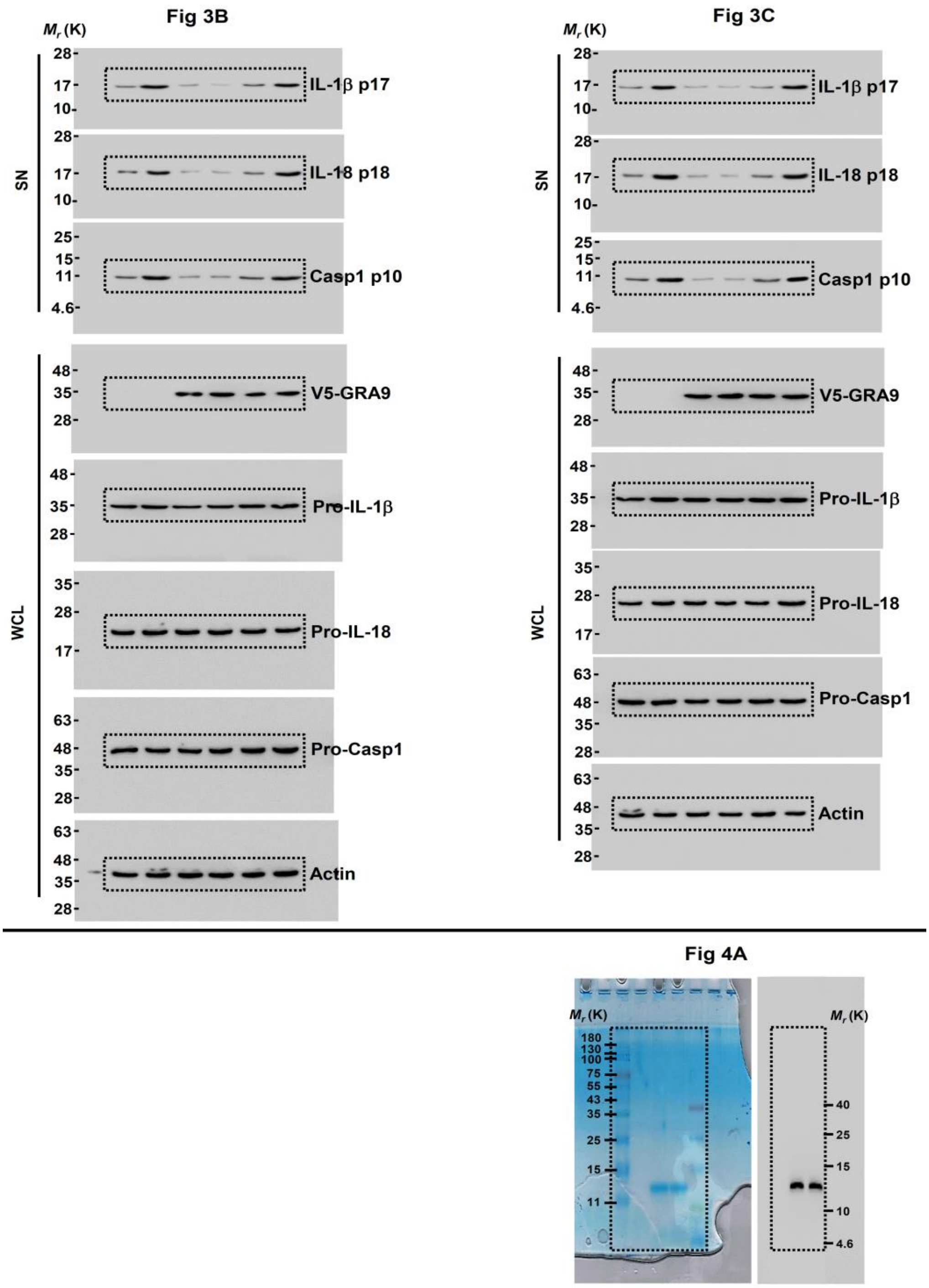

Figure S1. Full-length western blots.

Full-length images of the blots presented in the Figure 3B, 3C and 4A.

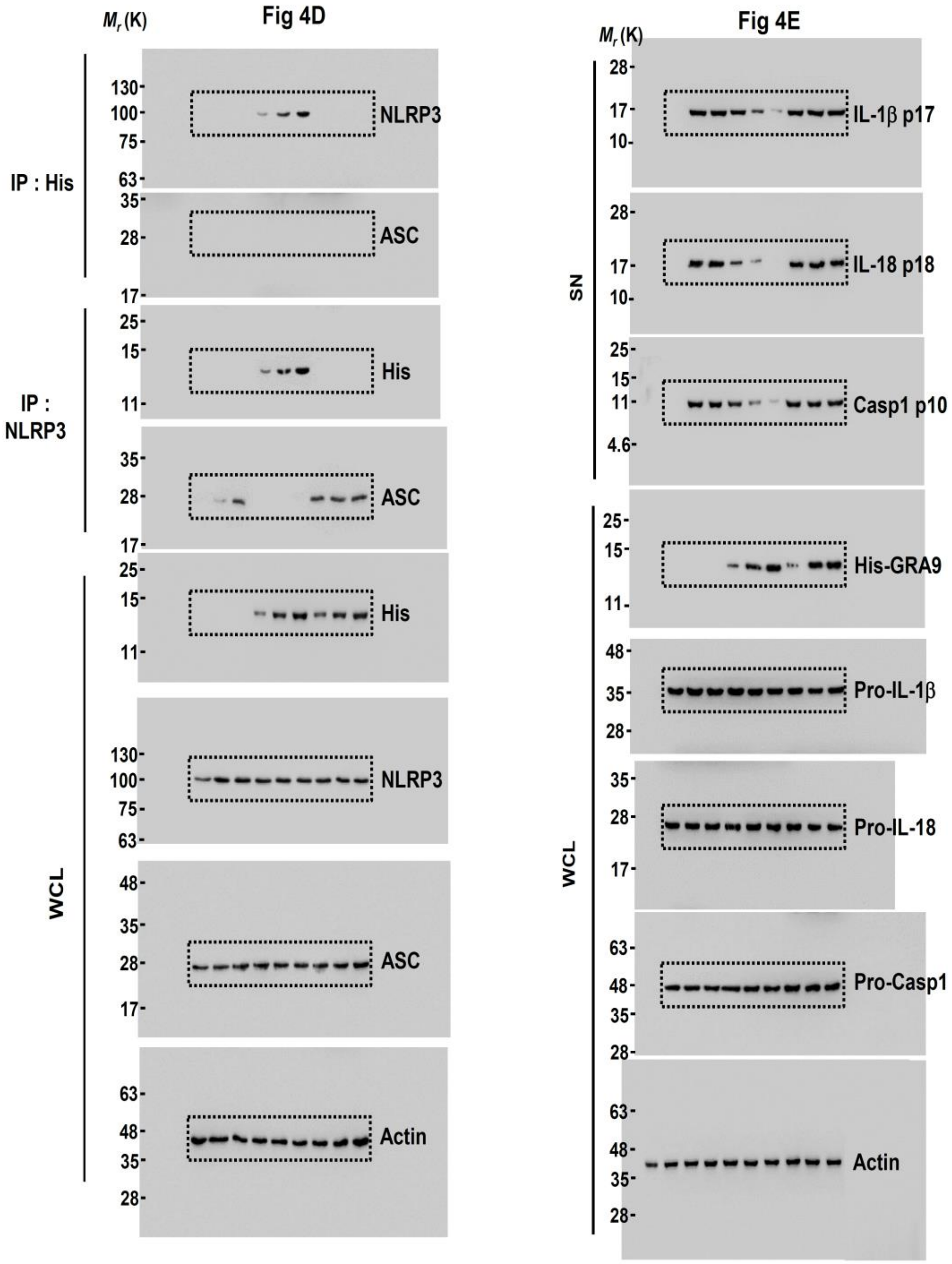

Figure S1. Full-length western blots.

Full-length images of the blots presented in the Figure 4D and 4E.

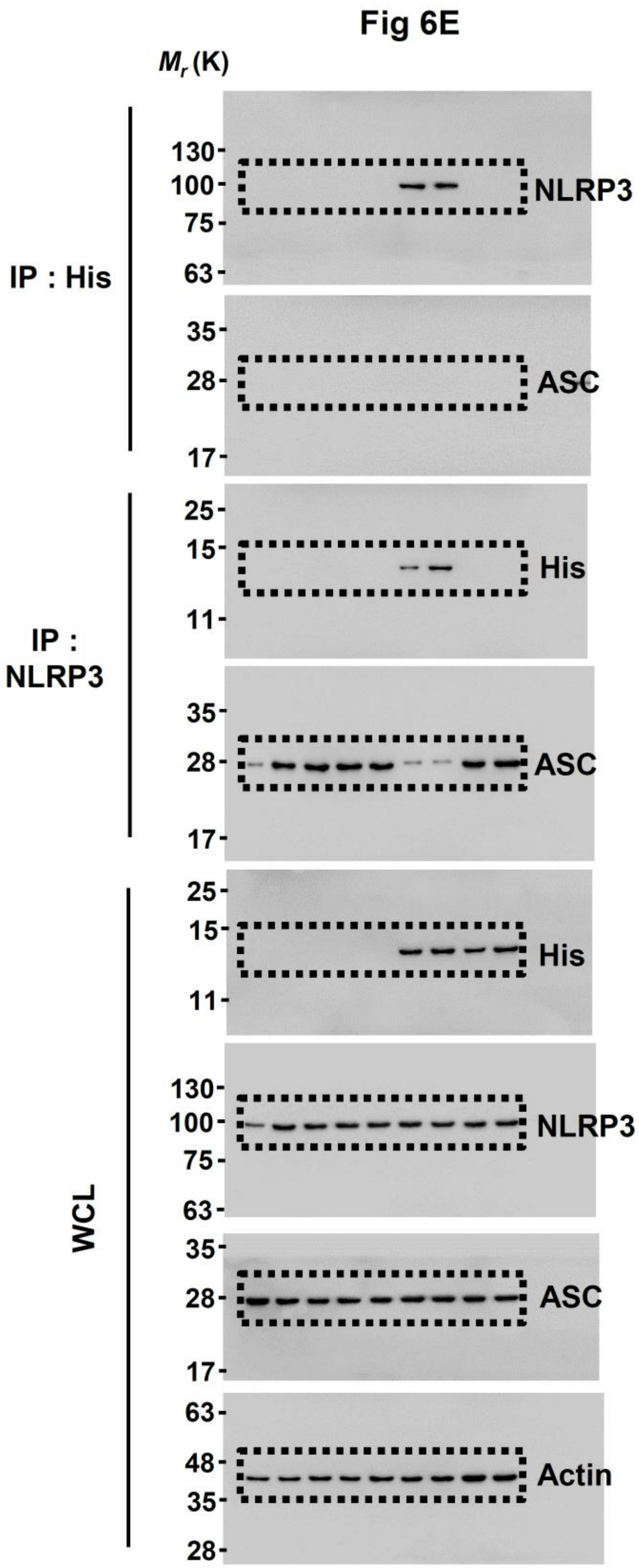

Figure S1. Full-length western blots.

Full-length images of the blots presented in the Figure 6E.

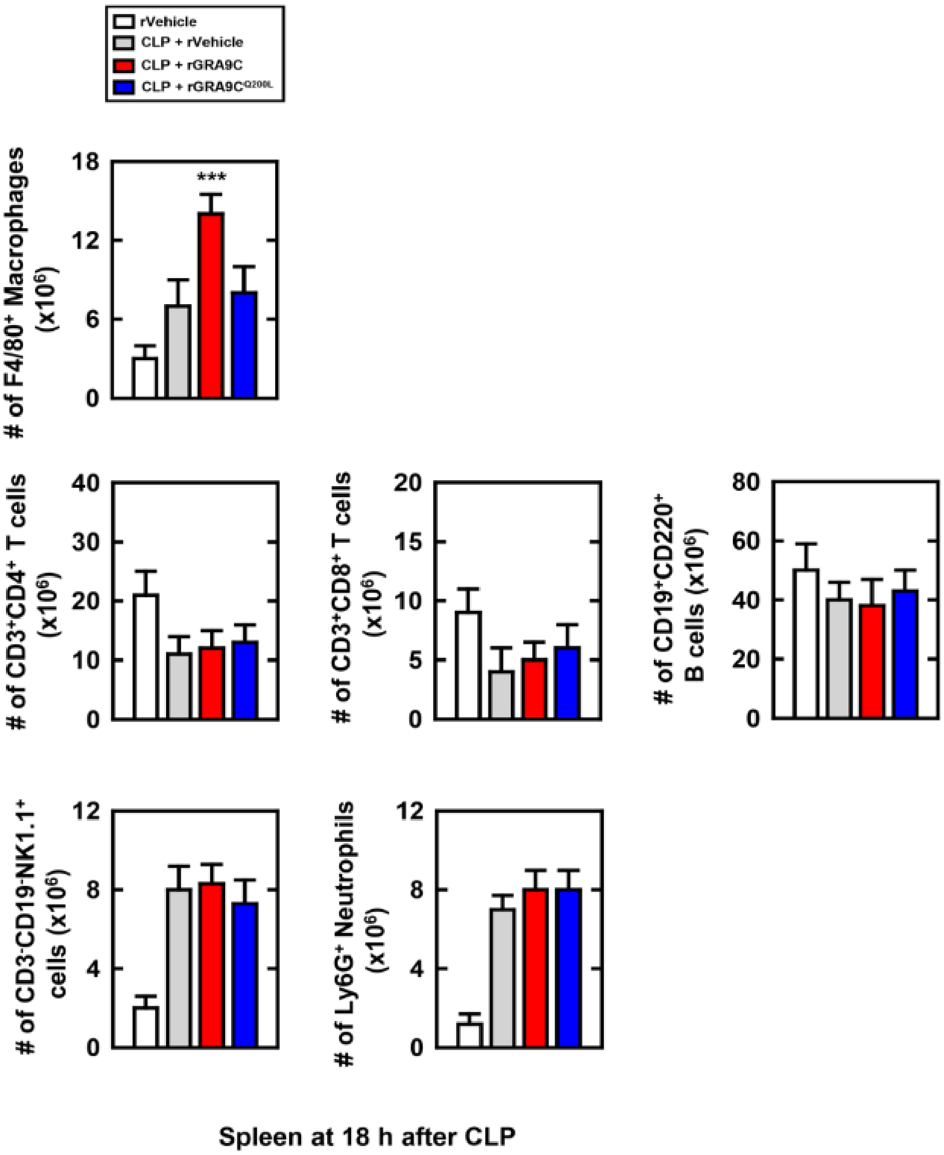

Figure S2. rGRA9C specifically increases the number of macrophages. The numbers of F4/80^{+} macrophages CD3^{+}CD4^{+} T cells, CD3^{+}CD8^{+} T cells, CD19^{+}CD220^{+} B cells, CD3^{-}CD19^{-}NK1.1^{+} cells and Ly6G^{+} Neutrophils found in the spleen using FACS analysis in the background CLP-induced sepsis after treatment of rVehicle, rGRA9C or rGRA9C^{Q200L} for 18 h. The data are representative of four independent experiments with similar results. Significant differences (*** $p < 0.001$) compared with rVector-treated mice.

Supporting Experimental Procedures

GST pulldown, immunoblot, and immunoprecipitation analysis

For GST pulldown, cells were harvested and lysed in NP-40 buffer supplemented with a complete protease inhibitor cocktail (Roche). After centrifugation, the supernatants were precleared with protein A/G beads at 4 °C for 2 h. Pre-cleared lysates were mixed with a 50% slurry of glutathione-conjugated Sepharose beads (Amersham Biosciences), and the binding reaction was incubated for 4 h at 4 °C. Precipitates were washed extensively with lysis buffer. Proteins bound to glutathione beads were eluted with SDS loading buffer by boiling for 5 min.

For immunoprecipitation, cells were harvested and then lysed in NP-40 buffer supplemented with a complete protease inhibitor cocktail (Roche). After pre-clearing with protein A/G agarose beads for 1 h at 4 ˚C, whole-cell lysates were used for immunoprecipitation with the indicated antibodies. Generally, 1–4 μg of commercial antibody was added to 1 ml of cell lysates and incubated

at 4°C for 8 to 12 h. After the addition of proteins A/G agarose beads for 6 h, immunoprecipitates were extensively washed with lysis buffer and eluted with SDS loading buffer by boiling for 5 min.

For immunoblotting, polypeptides were resolved by SDS-polyacrylamide gel electrophoresis (PAGE) and transferred to a PVDF membrane (Bio-Rad). Immuno detection was achieved with specific antibodies. Antibody binding was visualized by chemiluminescence (ECL; Millipore) and detected by a Vilber chemiluminescence analyzer (Fusion SL 3; Vilber Lourmat).

Protein purification and Mass spectrometry

To identify GRA9-binding proteins, THP-1 cells expressing Flag-GRA9 or vector were harvested and lysed with NP-40 buffer (50 mM HEPES, pH 7.4,150 mM NaCl, 1 mM EDTA, 1% (*v*/*v*) NP40) supplemented with a complete protease inhibitor cocktail (Roche, Basel, Switzerland). Post-centrifuged supernatants were precleared with protein A/G beads at 4 °C for 2 h. Precleared lysates were mixed with αFlag antibody-conjugated with agarose beads for 4 h at 4 °C. Precipitates were washed extensively with lysis buffer. Proteins bound to beads were eluted and separated on a Nupage 4–12% Bis-Tris gradient gel (Invitrogen). After silver staining (Invitrogen), specific protein bands were excised and analyzed by ion-trap mass spectrometry at the Korea Basic Science Institute (Seoul, Korea) Mass Spectrometry facility, and amino acid sequences were determined by tandem mass spectrometry and database searches.

Quantitative real-time polymerase chain reaction (PCR)

Total RNA was extracted from cells using an RNeasy RNA extraction Mini-Kit (Qiagen). cDNA was synthesized using an Enzynomix kit (Enzynomix) and quantitative PCR was performed using gene-specific primer sets (Bioneer) and SYBR Green PCR Master Mix (Roche). Real-time PCR was performed using a QuantStudio™ 3 (ABI), according to the manufacturer's instructions. Data were normalized to the expression of β-actin. Relative expression was calculated using the delta–delta Ct method. The sequences of the primers were as follows: mCD86 (Forward: gcacgtctaagcaaggtcac; Reverse: catatgccacacaccatccg), miNOS (Forward: ccccgctactactccatcag; Reverse: ccactgacacttcgcacaaa), mCD163 (Forward: tgtgaccatgctgaggatgt; Reverse: ctcgaccaatggcactgatg), mArg1 (Forward: ctgagctttgatgtcgacgg; Reverse: tcctctgctgtcttcccaag), mβ-Actin (Forward: aagtgtgacgttgacatc; Reverse: gatccacatctgctggaagg).

Confocal fluorescence microscopy

Immunofluorescence analysis was performed as described previously [32]. The cells were fixed on coverslips with 4% (*w*/*v*) paraformaldehyde in PBS and then permeabilized for 10 min using 0.25% (*v*/*v*) Triton X-100 in PBS at 25 ˚C. TRAF6 or His was detected using a 1/100 dilution of the primary Ab for 1 h at 25 ˚C. After washing, the appropriate fluorescently labeled secondary Abs were incubated for 1 h at 25 ˚C. Slides were examined using laser-scanning confocal microscopy (model LSM 800; Zeiss).

Cellular fractionation

Cytosol and mitochondria were isolated from cells using a Mitochondria Fractionation Kit (Active Motif, 40015) or as described previously [34]. Subcellular fractionated proteins were lysed in buffer containing 2% SDS and boiled with 2x reducing sample buffer for SDS-PAGE.

MTT assay

Cell viability relative to non-treated group was measured by MTT assay, as described previously [2]. After incubating for the indicated time points, 5 mg/ml of MTT (3-(4,5-dimethylthiazol-2-yl)-2,5-diphenyltetrazolium bromide) solution was added in the place of media, and cells were incubated for further 4 h. Then, all the media was removed and the same volume of dimethyl sulfoxide (DMSO) solution was added for 15 min to dissolve the formazan. Using UV/VIS spectrophotometer, each well of the plate was measured at 540 nm to measure relative cell viability.

Flow cytometry

Flow cytometry data were acquired on a FACSCanto (BD Biosciences, San Diego, CA) and analyzed with FlowJo software (Tree Star, Ashland, OR). To determine expression of cell surface proteins, mAb were incubated at 4˚C for 20–30 min and cells were fixed using Cytofix/Cytoperm Solution (BD Biosciences) and, in some instances followed by mAb incubation to detect intracellular proteins. The following mAb clones were used: NK1.1 (PK136, eBioscience), LY6G (1A8-Ly6g eBioscience), SR-A (PSL204, eBioscience), FcR (MAR-1, eBioscience), TLR2 (6C2, eBioscience), TLR4 (HTA125, eBioscience), NRP1 (3DS304M, eBioscience), CXCR2 (eBio5E8-C7-F10 (5E8-C7-F10), eBioscience).
